# The functional significance of vascular DNA hypermethylation in atherosclerosis: a historical perspective

**DOI:** 10.3389/fphar.2025.1562674

**Published:** 2025-04-15

**Authors:** Silvio Zaina

**Affiliations:** Department of Medical Sciences, Division of Health Sciences, Leon Campus, University of Guanajuato, Leon, Mexico

**Keywords:** atherosclerosis, epigenetic drug, DNA hypermethylation, epigenetics, therapy

## Abstract

A decade ago, independent mechanistic and descriptive epigenomics data demonstrated for the first time that vascular DNA hypermethylation is a landmark of and causal factor in human and murine atherosclerosis. Since then, a flurry of converging evidence has assigned a prominent role to vascular DNA hypermethylation across the natural history of cardiovascular disease (CVD), from the exposure to risk factors, to the onset and progression of the atheroma. DNA hypermethylation is induced by and mediates the metabolic outcomes of high-fat diets and CVD risk-enhancing lipids in several models. Early-stage atheroma DNA is hypermethylated compared to normal adjacent tissue, and that trend is amplified as the atheroma progresses. That evidence has resulted in a strong interest for epigenetic drugs in CVD. Crucially, the DNA methylation inhibitor azacytidine has been singled out as a potent guardian of the contractile, anti-atherogenic phenotype of smooth muscle cells (SMC). Those findings are gaining relevance, as the antiatherogenic effects of the anticancer drugs azacytidine and decitabine fit into the recently revived hypothesis that the atheroma is a SMC-driven cancer-like mass. Finally, this 10-year anniversary has been marked by the first report that nanoparticles loaded with a DNA methyltransferase inhibitor drug are anti-inflammatory and inhibit murine atherosclerosis. Exciting work lies ahead to assess whether DNA hypermethylation is a practical and effective target to prevent or cure human atherosclerosis.

## Introduction

This narrative review marks a decade of research that has established DNA hypermethylation as one of the central concepts in the epigenetics of the atheroma. The use of the general term “DNA hypermethylation” is justified by the fact that the data presented here have been obtained with biochemical inhibition or manipulation of the expression of DNA methyltransferases, rather than by targeting specific *loci*. A similar non-gene-specific strategy has been adopted in the CVD field by targeting transcription-permissive chromatin with inhibitors of the bromodomain and extraterminal-containing protein family (Borck et al., 2020). This “bull in a china shop” approach is not oblivious of the between-tissue and between-cell mosaicism of the DNA methylome within multicellular organisms, a phenomenon observed 5 decades ago (Waalwijk and Flavell, 1978). In the atheroma, epigenetic profiles are being discovered, that are specific for inflammatory, smooth muscle (SMC) and endothelial cells, and contribute to the transcriptional patterns that support the respective cell type’s function in atherosclerosis [see (Aziz et al., 2024) for a recent thorough review of the topic]. That rich cellular tapestry is being described in high detail by single cell transcriptomics and chromatin accessibility mapping. The number of identified cell types in the human atheroma is currently up to 25 (Örd et al., 2021; Turner et al., 2022; Zhang et al., 2022; Bashore et al., 2024). In particular, those studies have identified a plethora of SMC phenotypes including osteoblast-like, foam cell-like, fibromyoblast, proliferating and migrating. The topic has been extensively reviewed (Lambert and Jørgensen, 2024). Importantly, that new information enriches the traditional binary view of SMC phenotypes as either “synthetic” or “contractile” - proatherogenic or protective, respectively (Stavenow, 1984). At any rate, I will refer to the traditional SMC classification to reflect the content of the cited literature.

DNA hypermethylation first emerged as an alternative to the opposite view of the atheroma’s epigenetic landscape, *i.e.*, genome-wide loss of DNA methylation. As time progressed, a flurry of experimental and observational data have indicated that hypermethylation of a set of still incompletely identified *loci* plays a pivotal role in atherosclerosis. Marking a perspective-looking end of this decade, the first description of a nanoparticle-based strategy to bring the atheroma DNA methylation down to physiological levels has been published in 2024 (Márquez-Sánchez et al., 2024).

**FIGURE 1:**
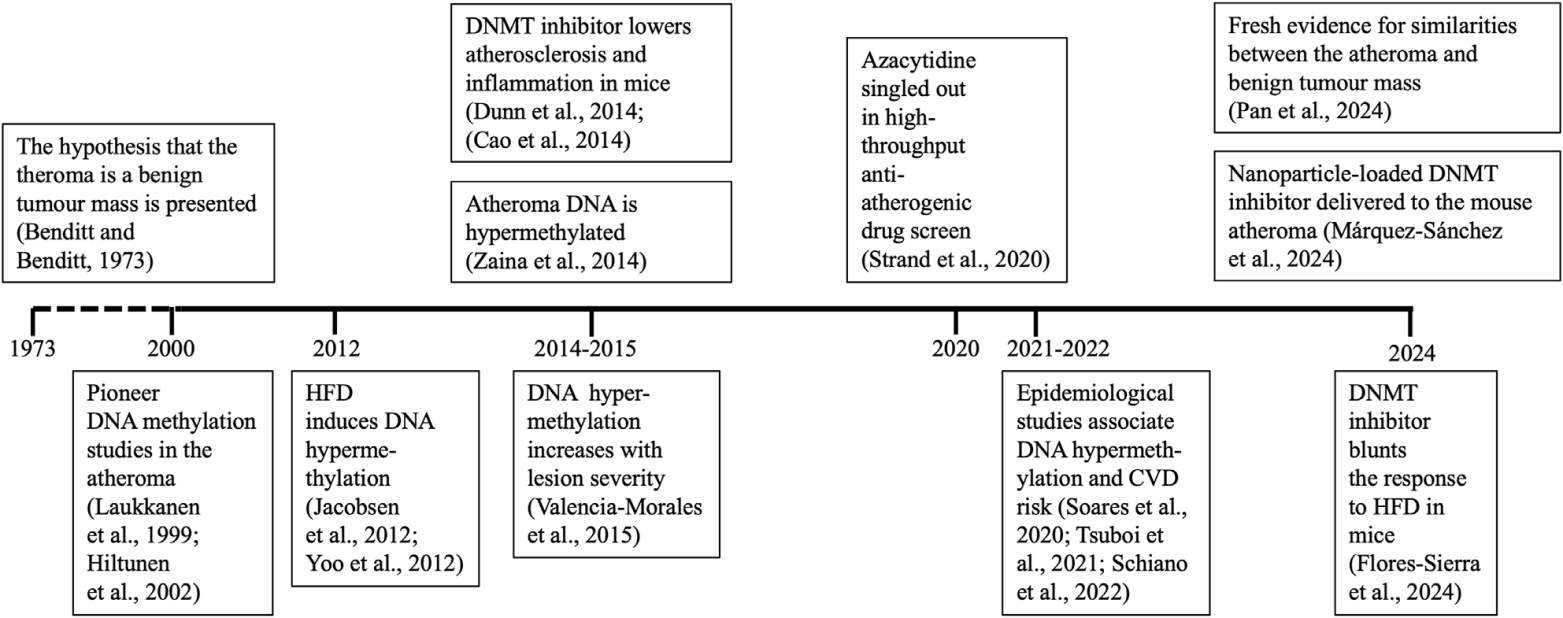
Timetable of relevant findings. Studies showing specific advances for the first time are shown. Positions of years on the time axis are not in scale. CVD, cardiovascular disease; DNMT, DNA methyltransferase. HFD, high-fat diet.

I will concentrate on atheroma DNA, therefore studies focused on the whole blood DNA methylome, which represent ∼95% of publications in cardiovascular epigenetics, will not be included in this review (Krolevets et al., 2023). Although I will focus on the last decade, reference to earlier work will provide the necessary historical context. A succinct timeline of the advances in the field is shown in Figure 1. I apologize for not including several important studies in that figure due to space limitations. Another necessary omission will by citing excellent work focused on gene-specific methylation.

## Is it DNA hypomethylation or hypermethylation?

Seminal studies in the 80 s initiated an intense effort to understand the DNA methylome of cancer, undoubtedly the best epigenetically understood disease (Greger et al., 1989). The more recent birth of cardiovascular epigenetics was marked by the observation that DNA hypomethylation was a feature of atherosclerosis: HPLC-based direct determination of 5-methylcytosine (5mdC) - the main product of DNA methylation in mammals - revealed a decrease in genome-wide DNA methylation in rabbit, mouse and human atherosclerotic arteries compared to non-atherosclerotic controls (Laukkanen et al., 1999; Hiltunen et al., 2002). Furthermore, those studies determined that the DNA of synthetic SMC was hypomethylated relative to the contractile counterparts. Those findings were independently confirmed using a range of techniques including nascent epigenomics approaches. A study based on sequencing of DNA fragments generated by methylation-sensitive amplification polymorphism analysis validated by biochemical assays, determined that the DNA of atherosclerotic murine aortas was hypomethylated compared to control mice (Lund et al., 2004). Genes and repeated elements were found to be hypomethylated, and the methylomes of the aortas of atherosclerotic and control mice diverged before any atheroma was histologically detectable. Later, a microarray-based interrogation of human CpG islands (CGI), reached comparable conclusions. CGI are relatively short genomic regions that are usually spared from the high levels of methylation found throughout the genome, and map to functional elements including housekeeping genes promoters (Bird et al., 1985). The study demonstrated that a significant subset of the few normally hypermethylated CGI in the vascular tissue were demethylated in coronary atherosclerosis in humans (Castillo-Díaz et al., 2010). Furthermore, DNA hypomethylation was detected of atherosclerotic femoral arteries relative to control mammary counterparts, and in atherosclerotic compared to healthy carotid arteries obtained from different human donors (Aavik et al., 2015; Greißel et al., 2015).

Almost concomitantly, data reaching opposite conclusions began to emerge. A study of the human lactate transporter monocarboxylate transporter 3 (*MCT3*) gene demonstrated that the DNA methyltransferase inhibitor azacytidine could lower *MCT3* promoter methylation and restore lactate intake to normal levels in human synthetic SMC (Zhu et al., 2005). This was a landmark study as it hinted that an unspecific inhibitor of the DNA methylation machinery could be anti-atherogenic, implying that hypermethylation of several *loci* in addition to the *MCT3* promoter was a causal factor in atherogenesis. In other words, the study for the first time defined both a potential epigenetic target and a simple way to hit that target to treat atherosclerosis. In 2014, the balance was decisively tilted towards DNA hypermethylation by independent experimental and descriptive studies. Disturbed blood flow induced the expression of DNA methyltransferase 1 (DNMT1) in the endothelium, and azacytidine reduced endothelial inflammation and atheroma size in mice (Dunn et al., 2014). Those data were replicated in human and swine counterparts (Jiang et al., 2014). The anti-inflammatory and anti-atherogenic effects of azacytidine were confirmed in a second mouse model, where the impact of that drug on macrophage migration and adhesion were detailed (Cao et al., 2014). Those studies also identified potential target genes. In addition, the advent of affordable DNA methylation microarrays and whole-genome sequencing allowed high-coverage epigenomics of atherosclerosis. The comparison of same-donor (paired) human vascular samples revealed that the DNA of the portion of the aorta occupied by the atheroma was hypermethylated across the whole genome relative to the adjacent histologically normal aortic tissue, independent of lesion stage (Zaina et al., 2014). The aorta data were validated in human carotid artery lesions. Genes targeted for hypermethylation were involved in SMC biology. Several of those *loci* replicated the findings of an independent DNA methylation microarray-based comparison of human atherosclerotic arteries with disease-free counterparts and veins (Nazarenko et al., 2011).

A flurry of confirming evidence across cardiovascular pathologies and models was published in the following years. DNA hypermethylation increased with lesion progression and targeted *loci* mostly involved in macrophage biology in humans (Valencia-Morales et al., 2015). Low expression of selected enzymes that drive DNA demethylation was detected in human peripheral artery disease and transplant vasculopathy compared to the respective controls (Zhao et al., 2018; Ostriker et al., 2021). DNA hypermethylation accompanied the development of the murine arterial wall neointima, a partial but useful surrogate of atherosclerosis (Li et al., 2020; Wang et al., 2020). Experimental studies consistently showed that the biochemical inhibition of DNA methylation or overexpression of enzymes involved in DNA demethylation mitigate atherosclerosis in animal models (Peng et al., 2016; Zhuang et al., 2017; Qu et al., 2022; Zhu et al., 2022). Furthermore, knocking out adenosine kinase in SMC mitigates aortic inflammation and induces a decrease in the availability of methyl group donors in mice (Xu et al., 2024). Additionally, azacytidine converts CD4^+^ T cells into regulatory T cells that inhibit atherosclerosis in a mouse model (Zhu et al., 2024). One of the most consequent pieces of evidence for cardiovascular epigenetics is the outcome of a large-scale (>3,000 compounds) molecular screen aiming at identifying novel antiatherogenic drugs in primary rat aortic SMC. Notably, the study singled out azacytidine as a promoter of the “contractile” phenotype of SMC. The mechanistic analysis of the response to azacytidine in SMC revealed that the maintenance of sustained expression of the phosphatase and tensin homolog - known as PTEN - was the underlying molecular phenomenon (Strand et al., 2020). Additionally, the study independently replicated several of the previously reported anti-inflammatory and anti-atherogenic responses elicited by azacytidine.

The controversy whether the DNA of the atheroma is hypomethylated or hypermethylated may have been settled, but its very existence begs for explanations. An important feature of the early studies of the DNA methylome of atherosclerosis is that diseased and control samples were obtained from different individuals or different vascular beds. It is therefore conceivable that the initial effects of the exposure to risk factors results in DNA hypomethylation - detectable in the normal portions of the arterial wall - and that DNA hypermethylation is imposed on the diseased portions of the same artery, but not to a sufficient extent to return to the physiological levels of DNA methylation of the arterial wall unexposed to risk factors. That model would reconcile inter-individual DNA hypomethylation and intra-vascular bed DNA hypermethylation; nonetheless, it is not compatible with the documented effects of cardiovascular risk factors on DNA methylation (see below). Rather, I submit that genetics and tissue-specificity are likely explanations. DNA methylation is under significant genetic control (Kerkel et al., 2008; Gunasekara et al., 2023). Also, it has been known for decades that the susceptibility to atherosclerosis differs across vascular tissue types and is associated with tissue-specific transcriptional and epigenetic profiles (Nazarenko et al., 2011; Brown, 2024). Interestingly, the human oestrogen receptor beta gene (*ESR2*) seems to be an exception in the described “unpaired versus paired” DNA methylation divergence. The methylation of *ESR2* promoter changed in the same direction in across-vasculature type (unpaired) or intra-individual same-vascular bed (paired) comparisons, suggesting a strictly atherosclerosis-specific epigenetic profile, irrespective of intra-individual or inter-individual epigenetic or genetic variation (Kim et al., 2007). It remains to be determined how many genes share that profile.

## Before the atheroma: cardiovascular risk factors and DNA hypermethylation

The literature mentioned in the previous section has uncovered epigenetic changes that were present in low histological grade lesions and therefore potentially represented early events that could predispose to atherosclerosis in humans (Zaina et al., 2014). Indeed, accumulating evidence has showed that DNA hypermethylation is imposed by cardiovascular risk factors before the onset of the atheroma. High-fat diets - prominent drivers of cardiovascular risk - induced DNA hypermethylation in a range of tissues - the liver, placenta, uterus, skeletal muscle, oocyte - in humans and rodents (Jacobsen et al., 2012; Yoo et al., 2012; Amaral et al., 2014; Yu et al., 2015; Seki et al., 2017; Ramaiyan and Talahalli, 2018; Bozdemir et al., 2024; Tang et al., 2024). Importantly, high-fat diet during the pre-pubertal period induces hepatic global DNA hypermethylation that resists subsequent feeding with control diet in a rat model, suggesting persistent epigenetic effects (Aguilar-Lozano et al., 2025). Lipids and fatty acids can affect either side of the same coin by increasing *de novo* DNA methylation and inhibiting the DNA demethylation pathway: excess dietary fat increased the expression of DNA methyltransferase in the murine ovary, testis and liver, while decreasing 5-hydroxy-methylcytosine (5hmdC) in mice (Pant et al., 2023; Sukur et al., 2023; Vasishta et al., 2024; Yang et al., 2024; Zhao et al., 2024). 5hmdC is the product of 5mdC oxidation by the ten-eleven translocation (TET) dioxygenases, and is an intermediate in the DNA demethylation pathway. Therefore, a decrease of 5hmdC is interpreted as stabilization of DNA’s methylated state. These effects have been observed also in a rat model of paternal transmission of high-fat diet-induced phenotypes (Haberman et al., 2024). Consistently, mutations of the member two of the TET family gene (*TET2*) observed in clonal haematopoiesis of indeterminate potential (CHIP) are associated with human atherosclerosis and respond to antiinflammatory therapy, whereas the outcome of DNA methyltransferase CHIP is less clear (Svensson et al., 2022). From a functional viewpoint, liver-specific loss of DNA methyltransferase activity counteracted the high-fat diet-induced glucose intolerance in mice (Yao et al., 2024). Also, the DNA methyltransferase inhibitor decitabine, an analogue of azacytidine, counteracted the metabolic effects of a high-fat diet in the skeletal muscle and liver in mice (Flores-Sierra et al., 2024). Those data were echoed by the reduction of liver fat content induced by azacytidine in a mouse model of exposure to high-fat diet and maternal overnutrition (Cheng et al., 2024). Comparable responses were obtained in experimental studies in human adipose tissue or cultured cells exposed to atherogenic lipoproteins or specific fatty acids (Rangel-Salazar et al., 2011; Silva-Martínez et al., 2016; Perfilyev et al., 2017). As *caveat*, recent evidence that loss of DNA methyltransferase activity in hematopoietic cells exacerbated the effects of a high-fat diet in mice, suggests that cell-specificity adds a layer of complexity in the interaction between the diet and the DNA methylome (Reyes et al., 2024). As for obesity, adipose tissue mesenchymal cells had net lower 5hmdC in human subjects with high BMI (Eirin et al., 2024). Furthermore, a ceramide cocktail reflecting the counterpart observed in humans with obesity induced DNA hypermethylation in cultured human THP-1 monocytes (Castro et al., 2019). Related to diabetes as cardiovascular risk, a high-glucose culture medium increases DNA methyltransferase expression in human umbilical vein endothelial cells (Vasishta et al., 2024). A study conducted in human aortic endothelial cells revealed robust responses to the challenge with increasing doses of glucose. Although CpG hypomethylation and hypermethylation were observed in comparable proportions, hypermethylation of the promoter of the vascular endothelial growth factor gene, a regulator of endothelial function, was observed (Pepin et al., 2021). Epidemiologically, CVD risk was associated with DNA hypermethylation in humans (Carla Silva Soares et al., 2020; Tsuboi et al., 2021; Schiano et al., 2022).

### How similar are the atheroma and cancer?

A striking aspect of the aforementioned evidence is that azacytidine and decitabine - two successful anti-tumour drugs - are anti-atherogenic. That notion is relevant in the light of the long history and recent revival of the hypothesis that the atherosclerotic lesion is a benign tumour-like mass. The idea is rooted in evidence published in the 70s that the human atheroma originates from clonal proliferation of SMC (Benditt and Benditt, 1973). The case for the epidemiological and functional overlap between the two diseases has been insightfully presented, and a common DNA methylation signature of CVD and cancer has been recently reported (Bell and Leeper, 2022; Domingo-Relloso et al., 2024). Accordingly, very recent work based on extensive multidisciplinary evidence has claimed that the atheroma is a tumour-like mass of proliferating SMC in humans and mice (Pan et al., 2024). The study also demonstrated the anti-atherogenic properties of yet another anti-cancer drug, niraparib. Incidentally, the anti-atherogenic activity of azacytidine or decitabine is generally overlooked in the literature. Although fascinating, the view of the atheroma as a tumour-like mass is challenged by inconsistent evidence. Pioneering epidemiological data showed that malignant tumours protect against atherosclerosis, and cardiovascular and cancer risk were inversely correlated at least in selected patient strata, contradicting some human studies (Wanscher et al., 1951; Bell and Leeper, 2022; Howe et al., 2022). Finally, inflammation is an often-cited link between cancer and atherosclerosis, yet it is involved in a plethora of different diseases and physiological responses (Furman et al., 2019; Cau and Saba, 2024).

**FIGURE 2:**
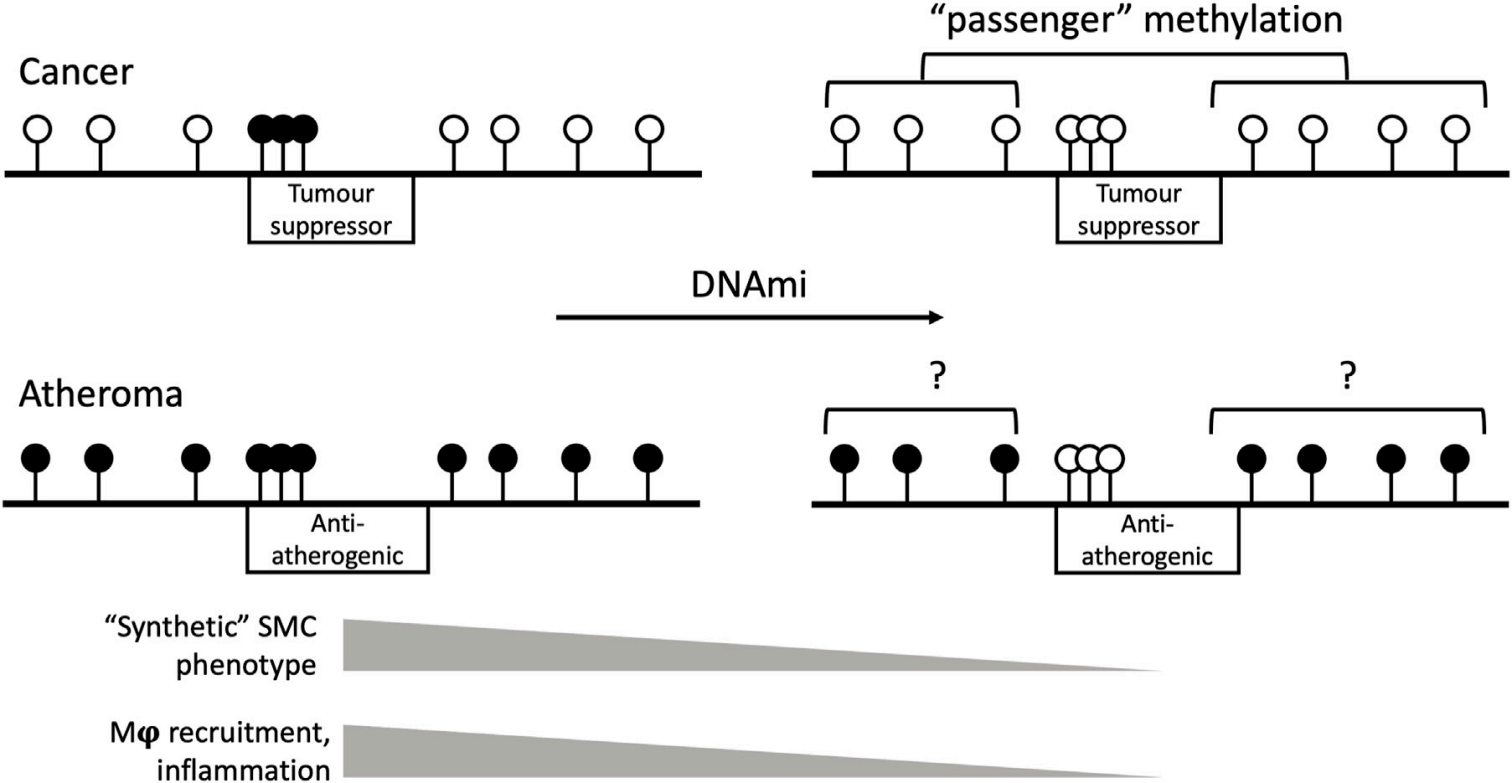
Schematic view of the genomic effects of DNA methyltransferase inhibitor drugs (DNAmi) in cancer and atherosclerosis. The horizontal black line represents the genome. Black and white lollipops indicate methylated and unmethylated sites, respectively. Boxes indicate the respective target gene type and their promoter methylation status. In cancer, the DNAmi azacytidine or decitabine target the hypermethylated promoters of tumour suppressor genes. The hypomethylation of the rest of the genome is functionally less relevant than local hypermethylation, hence the “passenger” term. In atherosclerosis, DNAmi improve SMC phenotype and inflammation by targeting anti-atherogenic gene promoters. The degree of selectivity and the effects of DNAmi on the background hypermethylated genome are poorly understood. Examples of likely anti-atherogenic targets are listed in Dunn et al., 2014; Cao et al., 2014. Mϕ and SMC, macrophage and smooth muscle cells, respectively.

Those inconsistencies notwithstanding, some valuable conclusions can be drawn from the clear anti-atherogenic effects of anti-cancer DNA methyltransferase inhibitor drugs. In general, human cancer DNA undergoes global hypomethylation and local hypermethylation notably in promoters of tumour suppressor genes (Kulis and Esteller, 2010). By contrast, genome-wide hypermethylation predominates in the human atheroma (Zaina et al., 2014). The available evidence suggests that hypermethylation of selected *loci* is not only mechanistically relevant in both diseases, but also a soft target for DNA methyltransferase inhibitor drugs, in contrast with the “passenger” nature of the surrounding DNA methylome (Figure 2). That interpretation is consistent with the non-random effects of azacytidine and decitabine, and the unintuitive strategy of curing cancer by inducing further hypomethylation of an already hypomethylated genome (Hagemann et al., 2011).

### Perspectives: therapeutic opportunities

Is DNA hypermethylation a worth pursuing therapeutic target to prevent or cure atherosclerosis? Cancer is again an important conceptual reference. The dream of cancer researchers is to achieve the erasure of the tumour mass by extensive cell death, but their cardiovascular colleagues have different concerns. Efficient cell death would likely lead to atheroma instability and acceleration of the very clinical complications that any cardiovascular therapy aims to avoid. The administration of azacytidine or decitabine - both nucleotide analogues - would therefore be counterproductive, as those drugs induce genomic instability and apoptosis as consequence of their incorporation into DNA (Hossain et al., 1997). Additionally, the significant side-effects of chemotherapy are a reasonable trade-off for cancer patients, but not for the bulk of cardiovascular counterparts. As further *caveat*, decitabine improves skeletal muscle mitochondrial function and hepatic steatosis, but induces adipose insulin resistance in high-fat diet-fed mice (Flores-Sierra et al., 2024). Yet, a carefully designed epigenetic intervention may offer a unique advantage, as it can reset the transcriptional program to force the reversion to a physiological phenotype, without any cell death. It is therefore imperative to implement drug delivery systems that leave the epigenome of non-target tissues unscathed. Excitingly, the last few years have witnessed promising advances in drug delivery to the vascular wall. Nanotechnology is the obvious reference, with a plethora of highly sophisticated carriers now available (Mao et al., 2023). In 2024, the effort to combine DNA methyltransferase inhibition and nanotechnology has walked a first step. Nanoparticles that were functionalized to bind to macrophage scavenger receptors, and loaded with the DNA methyltransferase inhibitor SGI-1027 lowered inflammation in cell culture and decreased atherosclerosis in a mouse model (Márquez-Sánchez et al., 2024). SGI-1027 is a non-nucleotide analogue; therefore it does not integrate into DNA and is less likely to promote the negative side effects of azacytidine or decitabine. Remarkably, RG108, another non-nucleotide DNA methylation inhibitor, lowers inflammation in a murine acute kidney injury model (Kong et al., 2024). A separate study showed that berberine-loaded nanoparticles slowed atherosclerosis in mice (Wu et al., 2024). The plant alkaloid berberine has anti-cancer activity and can inhibit DNA methyltransferase expression in human cultured cancer cells (Qing et al., 2014). Further research will show whether this budding area of experimental cardiovascular medicine will grow into a success for patients.
